# Cardiovascular risk associated with intellectual disability among adults with type 2 diabetes: a nationwide cohort study in South Korea

**DOI:** 10.1016/j.eclinm.2026.104043

**Published:** 2026-07-01

**Authors:** Jong-Ha Baek, Yong-Moon Mark Park, Ga Eun Nam, Oak-Kee Hong, Ejin Park, Seung-Hyun Ko, Seong-Su Lee, Kyungdo Han

**Affiliations:** a Department of Epidemiology, Fay W. Boozman College of Public Health, University of Arkansas for Medical Sciences, Little Rock, AR, USA; b Department of Internal Medicine, Gyeongsang National University Changwon Hospital, Gyeongsang National University College of Medicine, Changwon, South Korea; c Cancer Prevention and Population Sciences Research Program, Winthrop P. Rockefeller Cancer Institute, University of Arkansas for Medical Sciences, Little Rock, AR, USA; d Department of Family Medicine, Korea University Guro Hospital, Korea University College of Medicine, Seoul, South Korea; e Department of Internal Medicine, College of Medicine, The Catholic University of Korea, Seoul, South Korea; f Department of Psychiatry, Incheon St. Mary's Hospital, College of Medicine, The Catholic University of Korea, Seoul, South Korea; g Division of Endocrinology and Metabolism, Department of Internal Medicine, St. Vincent's Hospital, College of Medicine, The Catholic University of Korea, Seoul, South Korea; h Department of Statistics and Actuarial Science, Soongsil University, Seoul, South Korea

**Keywords:** Intellectual disability, Type 2 diabetes, Cardiovascular disease, Myocardial infarction, Ischemic stroke

## Abstract

**Background:**

Individuals with intellectual disability (ID) are at elevated risk for cardiovascular disease (CVD), a vulnerability compounded by coexisting type 2 diabetes (T2D). We evaluated the extent to which ID is associated with incident CVD by directly comparing ID with both non-ID disabilities and no disabilities.

**Methods:**

We conducted a nationwide, population-based cohort study of adults with T2D (aged ≥20 years) in South Korea, with health screenings between January 1, 2015, and December 31, 2016, and follow-up until December 31, 2022 (median 5.9 years). Individuals with a prior myocardial infarction (MI) or ischemic stroke, missing covariates, or a cardiovascular event within the first year of follow-up were excluded. Disability was classified as ID, non-ID disabilities, or no disabilities using the Korea National Disability Registration System. The primary outcome was incident CVD, defined as MI (ICD-10: I21–22) or ischemic stroke (ICD-10: I63–64), assessed throughout follow-up. Hazard ratios (HRs) with 95% confidence intervals (CIs) were estimated using Cox proportional hazards models.

**Findings:**

Of 2,062,821 adults with T2D, 4574 (0.22%) had ID, 173,350 (8.4%) had non-ID disabilities, and 1,884,897 (91.4%) had no disabilities, and 111,450 incident CVD events were recorded during follow-up. The ID group had the highest CVD risk compared to those with no disabilities (HR 1.72; 95% CI 1.52–1.94), substantially exceeding non-ID disabilities (HR 1.35; 95% CI 1.33–1.37; *P* < .001). This vulnerability was most pronounced for ischemic stroke (HR 1.91; 95% CI 1.62–2.25), and remained evident even in mild ID (HR 1.61; 95% CI 1.36–1.91).

**Interpretation:**

ID is associated with a substantially greater CVD risk burden in adults with T2D than other disabilities, particularly for ischemic stroke. Future studies are needed to evaluate long-term cardiovascular trajectory and targeted prevention strategies in this population.

**Funding:**

This study was supported by the 10.13039/501100002701Ministry of Education of the Republic of Korea and the 10.13039/501100003725National Research Foundation of Korea (K.H.; NRF-2023S1A5A2A21083821). This research was also supported by the GLAMP Program of the 10.13039/501100003725National Research Foundation of Korea (NRF) grant funded by the Ministry of Education (No. RS-2025-25441317).

**Research in context**

**Evidence before this study**

We searched PubMed and Embase for articles published from database inception to June 30, 2025, using the terms “intellectual disability”, “type 2 diabetes”, and “cardiovascular disease”, without language restrictions. We included population-based observational studies and systematic reviews reporting cardiovascular outcomes in individuals with intellectual disability. Studies were excluded if they did not report original cardiovascular outcome data or lacked a comparator group. The overall quality of evidence was limited by heterogeneous study populations, restricted age ranges, and the absence of direct comparisons across disability subtypes. A recent systematic review comprehensively synthesized the prevalence and incidence of cardiovascular disease in adults with intellectual disability (ID) and highlighted substantial heterogeneity across prior studies. Existing primary studies were conducted in restricted age groups, including children and adolescents (aged <20 years) and adults aged 50 or older. Prior studies rarely compared ID with other disability categories, leaving it unclear whether any excess cardiovascular risk is unique to ID or reflects a generalized disability-related vulnerability. Population-based data specifically quantifying cardiovascular outcomes in individuals with comorbid type 2 diabetes and ID remain notably scarce.

**Added value of this study**

This study, based on nationwide population-based data from over two million individuals with type 2 diabetes in South Korea, provides a comprehensive evaluation of cardiovascular disease risk associated with ID. Using a group with no disabilities as a reference, individuals with ID presented the highest risk for overall cardiovascular disease, especially exceeding the risk observed in the non-ID disability group. Notably, this elevated risk persisted after additional adjustment for traditional cardiometabolic risk factors and in analyses that accounted for the competing risk of mortality. The vulnerability was most pronounced for ischemic stroke. Furthermore, although risk estimates were higher with greater ID severity, elevated risk was already evident in individuals with mild ID. Finally, no significant difference in cardiovascular risk associated with ID was observed across the age spectrum, indicating a lifelong vulnerability.

**Implications of all the available evidence**

Although ID is recognized as a risk factor for cardiovascular disease, this study demonstrated that its associated risk burden significantly exceeds that of other disability types. This elevated risk remained robust among individuals with type 2 diabetes even after additional adjustment for cardiometabolic factors and in competing-risk analyses. Notably, the distinct vulnerability to ischemic stroke, observed within a relatively short follow-up and evident even in mild ID, suggests that conventional cardiovascular risk prevention strategies alone may be insufficient. These findings support more proactive, multidisciplinary care models that integrate targeted stroke prevention across the entire ID severity spectrum. Future longitudinal studies with extended follow-up and intervention-based research are warranted to clarify the long-term cardiovascular trajectory of individuals with ID and type 2 diabetes and evaluate the effectiveness of targeted prevention and multidisciplinary care strategies in this population.

## Introduction

Individuals with type 2 diabetes (T2D) are at high risk for cardiovascular disease (CVD). Previous population-based studies suggest that individuals with intellectual disability (ID) have a higher risk of T2D and may develop T2D at younger ages than the general population.^1^ This burden is often compounded for those living with disabilities, who not only face a greater prevalence of cardiometabolic risk factors^2^, ^3^, ^4^ but also encounter significant systemic barriers to optimal healthcare.^5^, ^6^, ^7^, ^8^ Indeed, individuals with disabilities often experience a premature onset of chronic health conditions, irrespective of their primary impairment.^9^

In particular, individuals with ID have a greater prevalence of T2D^10^ and face unique challenges such as cognitive impairments, shorter clinical encounters, and difficulties in patient–provider communication, further hindering optimal diabetes self-management in this population.^11^^,^^12^ Consequently, individuals living with ID may be at particularly high risk for CVD and related complications compared with the general population.^13^^,^^14^

Despite this presumed vulnerability, population-based data specifically quantifying the magnitude of CVD risk in individuals with comorbid T2D and ID remain scarce.^15^ In addition, prior studies in the general ID population have yielded inconsistent findings, with some reporting increased CVD risk in younger individuals^13^^,^^16^ and others reporting no significant associations in older adults (aged ≥50 years).^17^, ^18^, ^19^ Moreover, previous investigations rarely compared ID with other disability categories, leaving uncertainty about whether any excess CVD risk is unique to ID or simply reflects the generalized risk associated with living with any disability. This distinction is important for moving beyond risk identification toward understanding a critical health disparity.

To address these knowledge gaps, we conducted a nationwide, population-based cohort study with data from the South Korean National Health Insurance Service (NHIS) to evaluate the association of ID with the overall risk of CVD and CVD subtypes in individuals with T2D. By encompassing a broad age spectrum and utilizing a nationwide sample, this study provides a comprehensive evaluation of CVD risk in the understudied population of individuals with comorbid ID and T2D.

## Methods

### Study design and setting

**Fig. 1:**
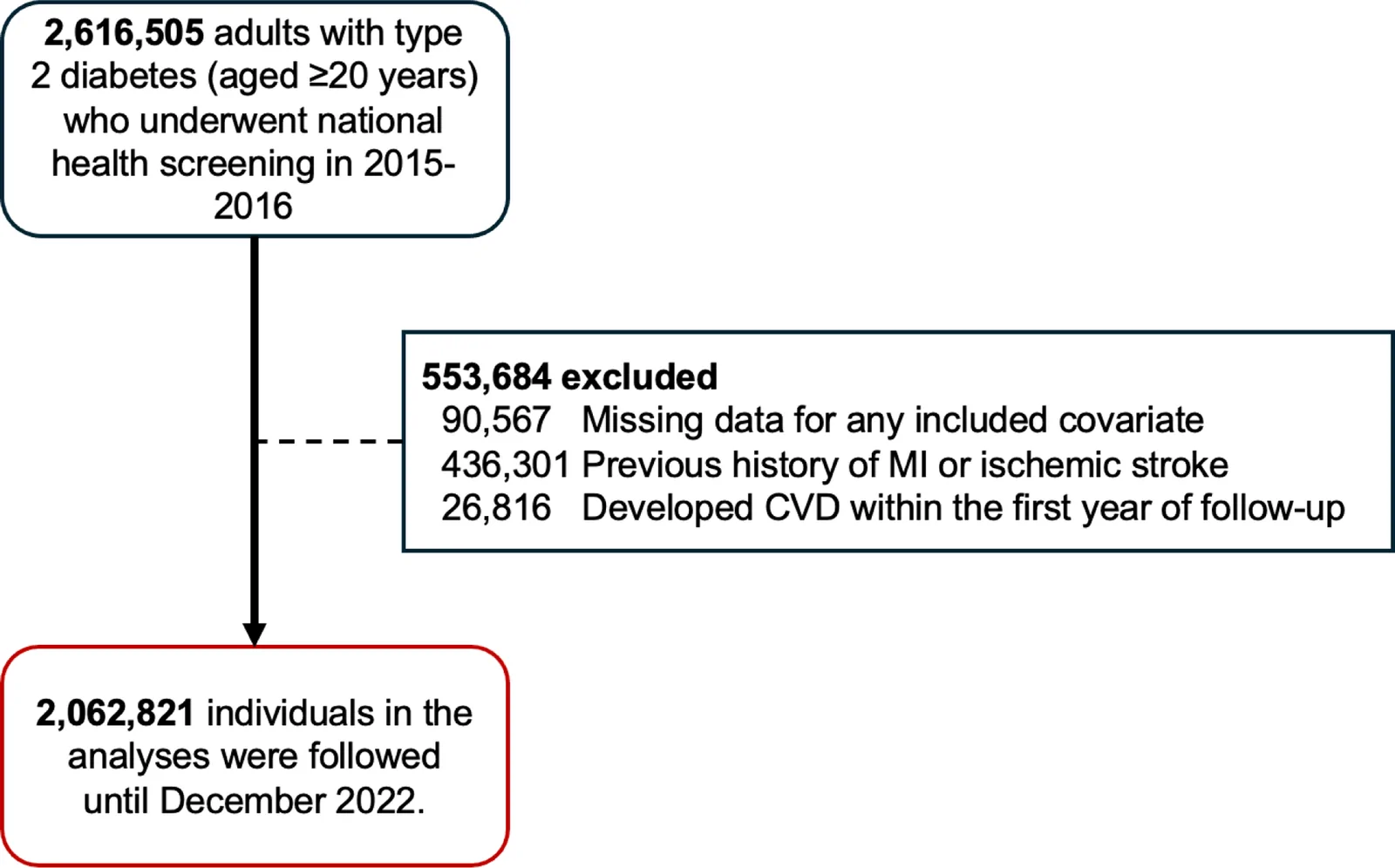
**Study profile**.

This nationwide, population-based observational cohort study included 2,616,505 individuals with T2D who underwent standardized general health screenings provided by the NHIS between January 1, 2015, and December 31, 2016. The 2015–2016 baseline period was selected to capture one full NHIS health-screening cycle with standardized baseline variables and to allow adequate follow-up for incident cardiovascular events. This period also preceded the 2019 KNDRS reform, helping preserve consistency in disability classification and severity grading. For individuals who underwent multiple health screenings during the inclusion period, the earliest screening date was designated the index date (baseline). The study population was restricted to adults aged 20 years or older to minimize the inclusion of individuals with type 1 diabetes. Also, to minimize the omission of undiagnosed T2D cases at baseline, we did not rely solely on ICD-10 diagnostic codes. Instead, our operational definition of T2D required either relevant ICD-10 codes for T2D (E11–14) combined with at least one insurance claim per year for prescribed antidiabetic medication, or a high fasting plasma glucose level (≥126 mg/dL) identified during the national health screening.^20^ Individuals with type 1 diabetes, as indicated by the ICD-10 code E10, or gestational diabetes mellitus, as indicated by the ICD-10 code O24, were excluded. Individuals were also excluded if they had missing data for any covariates (n = 90,567) and a previous history of myocardial infarction (MI) or ischemic stroke before the index date (n = 436,301). Additionally, individuals who developed any CVD diagnosis within their first year of follow-up were excluded to minimize reverse causality and potential bias related to undetected preexisting CVD (n = 26,816). Finally, 2,062,821 individuals with T2D were included in the final analytic cohort (Fig. 1).

### Data sources

We used data from the South Korean NHIS, a universal, single-payer public healthcare system that covers approximately 97% of the South Korean population (∼50 million individuals), while the remaining population is covered by the Medical Aid program integrated into the national claims system. Detailed descriptions of the NHIS database have been published elsewhere.^21^ In brief, the NHIS contains comprehensive claims-based information, including demographic characteristics, diagnostic codes based on the International Classification of Diseases, Tenth Revision (ICD-10), healthcare utilization, and prescription records. In addition, the NHIS is linked to the national general health screening program, which is administered biennially or prior to employment and provides standardized assessments of anthropometric, laboratory, imaging, and behavioral data. Additionally, information on disability status was obtained through linkage with the Korea National Disability Registration System (NDRS), which is integrated into the NHIS database. The validity of the NHIS database for epidemiologic research, as well as the operational definitions of T2D and major cardiovascular outcomes, has been supported by previous South Korean studies.^20^^,^^22^^,^^23^

The NDRS classifies disabilities into 15 legally defined subtypes encompassing physical, sensory, internal organ, psychiatric, and intellectual impairments. Because NDRS registration confers significant social welfare benefits, it requires a formal medical evaluation by a certified specialist, supported by medical records and functional assessment to confirm the diagnosis and functional impairment. Each disability subtype is graded by severity (grades 1–3), based on functional loss.^24^ In the NDRS, individuals with multiple concurrent disabilities may have both primary and secondary registered disabilities. However, because the NHIS research database provides only the primary registered disability variable, classification into the ID or non-ID disability groups was based on that variable.

### Definition of the severity of ID and classification of disability types

The disability grading process requires standardized intelligence quotient (IQ) testing and comprehensive functional assessments administered by certified specialists in psychiatry, neurology, or rehabilitation medicine. This rigorous evaluation supports the validity of the ID variable. The severity of ID was categorized into two levels: moderate-to-severe ID (grade 1, IQ < 35; grade 2, IQ 35−49) and mild ID (grade 3, IQ 50−69). To investigate the association between ID and CVD risk, individuals were stratified into three groups to compare CVD risk by disability status: 1) those with ID, 2) those with non-ID disabilities, and 3) those without any registered disabilities.

### Ascertaining study outcomes and covariates

The primary outcome was the first occurrence of any CVD, identified by ICD-10 codes and defined as hospitalization for MI (I21−22) or ischemic stroke (I63−64); ischemic stroke additionally required claims for brain imaging (computed tomography or magnetic resonance imaging) during follow-up. For subtype analyses, the incidence of MI and ischemic stroke was independently tracked from the date of the first event. Follow-up for incident CVD events continued through December 31, 2022.

Baseline covariates included sociodemographic variables (age, sex, income, and residence) and lifestyle behaviors (smoking, alcohol, and physical activity), collected through standardized, self-administered questionnaires completed by participants during the national health screening. Furthermore, we assessed cardiometabolic comorbidities (body mass index [BMI], hypertension, dyslipidemia, and chronic kidney disease [CKD]), and diabetes-related factors (diabetes duration, history of insulin prescription, use of triple combination antidiabetic drugs, and fasting plasma glucose). Information on biological sex was determined based on administrative resident registration data within the KNHIS database. Detailed operational definitions and classification criteria for all variables are provided in the Supplementary Method. Race and ethnicity data were not available in the KNHIS database and, therefore, were not analyzed.

### Statistical analysis

As this study utilized a nationwide administrative database encompassing the entire eligible population of adults with T2D who underwent national health screenings, formal a priori sample size calculation was not performed. Detailed inclusion and exclusion criteria are described in the Study design and Setting section above. Baseline characteristics are presented as percentages or means with standard deviations (SD). The incidence of CVD is reported per 1000 person-years.

The primary outcome was incident CVD, defined as the first occurrence of MI or ischemic stroke. Secondary outcomes included MI and ischemic stroke assessed independently. Cox proportional hazards regression models were used to estimate hazard ratios (HRs) and 95% confidence intervals (CIs) as the primary analytical approach, given the time-to-event nature of the outcome data. The proportional hazards assumption was verified using Schoenfeld residuals and the log-minus-log survival plots, with no significant violations observed. Tests for linear trends were performed by treating ordinal categories of disability severity and disability type as continuous variables.

To account for potential confounders, we constructed incremental multivariable models. Following Model 1 (unadjusted) and Model 2 (age and sex), Model 3 further adjusted for socioeconomic factors (low-income status and residential location),^22^ and served as our primary model to estimate the total association between ID and CVD risk. Subsequent fully adjusted analyses (Models 4 and 5) incorporated lifestyle-related behaviors, cardiometabolic comorbidities, and diabetes-related factors to explore potential mediating pathways.

Stratified analysis and a likelihood ratio test were performed to evaluate potential effect modifications. In a pre-specified sensitivity analysis, we categorized the 15 specific disability subtypes into four broad subgroups to compare the relative CVD risk magnitudes: 1) impairments involving external body functions (impairments of the extremities, vision, hearing, speech and language, disabilities due to brain injury, and facial deformities); 2) impairments involving internal body functions (renal failure, heart problems, liver disease, respiratory problems, ostomy, and epilepsy); 3) psychiatric disabilities other than ID (autism and mental disorders); and 4) ID. As post-hoc analyses conducted in response to peer review, we additionally performed a Fine–Gray subdistribution hazards model to account for the competing risk of all-cause mortality and a sensitivity analysis combining individuals with autism and ID into a single neurodevelopmental disorder group, given their frequent co-occurrence. Statistical analyses were performed with SAS, version 9.4 (SAS Institute Inc., Cary, NC, USA). The *P* values are 2-sided, and statistical significance was set at 0.05.

### Ethics

This study was approved by the Institutional Review Board (IRB) of Soongsil University, Seoul, South Korea (reference number SSU-202303-HR-494-1). The requirement for written informed consent was waived by the IRB owing to the retrospective design of the study and the use of pre-existing, de-identified administrative data from the South Korean NHIS. This study was conducted in accordance with the ethical principles outlined in the Declaration of Helsinki.

### Role of the funding source

The funders had no role in study design, data collection, data analysis, data interpretation, or writing of the report.

## Results

Among the 2,062,821 individuals with T2D, 1,884,897 (91.4%) had no disabilities, 173,350 (8.4%) had non-ID disabilities, and 4574 (0.22%) had an ID. Baseline characteristics of the study population are presented in Table 1 and Supplementary Table S1. The non-ID disability group was the oldest (mean age, 63.2 years) and exhibited the highest prevalence of hypertension (63.5%), dyslipidemia (56.6%), and CKD (14.8%). Conversely, the ID group was the youngest (mean age, 48.7 years). Despite having the highest prevalence of low-income status (78.1%) and obesity (54.4%), the ID group showed the lowest prevalence of hypertension (44.6%), dyslipidemia (47.2%), and CKD (5.5%), consistent with its younger age distribution. Furthermore, while individuals with ID reported the lowest rates of smoking and alcohol consumption, they also had the lowest levels of regular physical activity (15.6%). Regarding diabetes management, both the ID and non-ID disability groups were more frequently prescribed insulin and triple combination antidiabetic therapy compared to those without disabilities.

**Table 1:** Baseline characteristics of the study population by disability status.

N	Total	Disability status		
		No disabilities	Non-ID disabilities	ID
	2,062,821	1,884,897	173,350	4574
Percentage (n, %)				
Age groups, years				
20 to <40	118,865 (5.8)	115,622 (6.1)	2504 (1.4)	739 (16.2)
40 to <65	1,345,956 (65.3)	1,250,241 (66.3)	92,097 (53.1)	3618 (79.1)
≥65	598,000 (29.0)	519,034 (27.5)	78,749 (45.4)	217 (4.7)
Sex, male	1,266,693 (61.4)	1,155,440 (61.3)	108,770 (62.8)	2483 (54.3)
Sex, female	796,128 (38.6)	729,457 (38.7)	64,580 (37.2)	2091 (45.7)
Low-income status, yes^a^	441,875 (21.4)	388,235 (20.6)	50,068 (28.9)	3572 (78.1)
Residential location				
Urban	907,786 (44.0)	835,494 (44.3)	70,879 (40.9)	1413 (30.9)
Rural	1,155,035 (56.0)	1,049,403 (55.7)	102,471 (59.1)	3161 (69.1)
Smoking				
Non-smoker	1,104,671 (53.6)	1,003,473 (53.2)	97,477 (56.2)	3721 (81.4)
Ex-smoker	461,840 (22.4)	422,018 (22.4)	39,556 (22.8)	266 (5.8)
Current smoker	496,310 (24.1)	459,406 (24.4)	36,317 (21.0)	587 (12.8)
Alcohol consumption				
None	1,128,135 (54.7)	1,012,924 (53.7)	111,225 (64.2)	3986 (87.1)
Mild	730,645 (35.4)	682,540 (36.2)	47,617 (27.5)	488 (10.7)
Heavy	204,041 (9.9)	189,433 (10.1)	14,508 (8.4)	100 (2.2)
Regular exercise, yes^b^	452,525 (21.9)	416,804 (22.1)	35,034 (20.2)	687 (15.0)
Duration of diabetes				
Newly diagnosed	691,344 (33.5)	650,411 (34.5)	39,440 (22.8)	1493 (32.6)
<5 years	499,578 (24.2)	458,539 (24.3)	39,762 (22.9)	1277 (27.9)
5 to <10 years	416,049 (20.2)	373,857 (19.8)	41,042 (23.7)	1150 (25.1)
≥10 years	455,850 (22.1)	402,090 (21.3)	53,106 (30.6)	654 (14.3)
Insulin prescription, yes	161,385 (7.8)	138,791 (7.4)	22,165 (12.8)	429 (9.4)
Use of triple-combination drugs, yes	462,091 (22.4)	417,840 (22.2)	43,130 (24.9)	1121 (24.5)
Hypertension, yes	1,117,820 (54.2)	1,005,645 (53.4)	110,133 (63.5)	2042 (44.6)
Dyslipidemia, yes	1,123,562 (54.5)	1,023,286 (54.3)	98,117 (56.6)	2159 (47.2)
Obesity, yes	1,061,936 (51.5)	970,774 (51.5)	88,673 (51.2)	2489 (54.4)
Chronic kidney disease, yes	166,186 (8.1)	140,229 (7.4)	25,705 (14.8)	252 (5.5)
Mean (SD)				
Age, years	58.0 ± 11.8	57.6 ± 11.8	63.2 ± 10.7	48.7 ± 10.6
BMI, kg/m^2^	25.4 ± 3.6	25.4 ± 3.6	25.3 ± 3.6	25.9 ± 4.8
Blood pressure, mm Hg				
Systolic	128 ± 15	128 ± 15	129 ± 15	125 ± 16
Diastolic	78 ± 10	78 ± 10	78 ± 10	78 ± 11
FPG, mg/dL	146 ± 46	146 ± 46	141 ± 45	149 ± 65
Total cholesterol, mg/dL	188 ± 44	189 ± 44	181 ± 42	186 ± 44
LDL-cholesterol, mg/dL	105 ± 38	106 ± 39	100 ± 37	105 ± 38

Abbreviations: ID, intellectual disability; SD, standard deviations; FPG, fasting plasma glucose; LDL, low-density lipoprotein.

Values show mean ± SD unless otherwise indicated.

a Low household income status was defined as being in the lowest income quartile based on health insurance premiums or receiving Medical Aid benefits.

b Regular exercise was defined to be at least 30 min of moderate physical activity for ≥5 days weekly or at least 20 min of strenuous physical activity ≥3 days weekly.

**Fig. 2:**
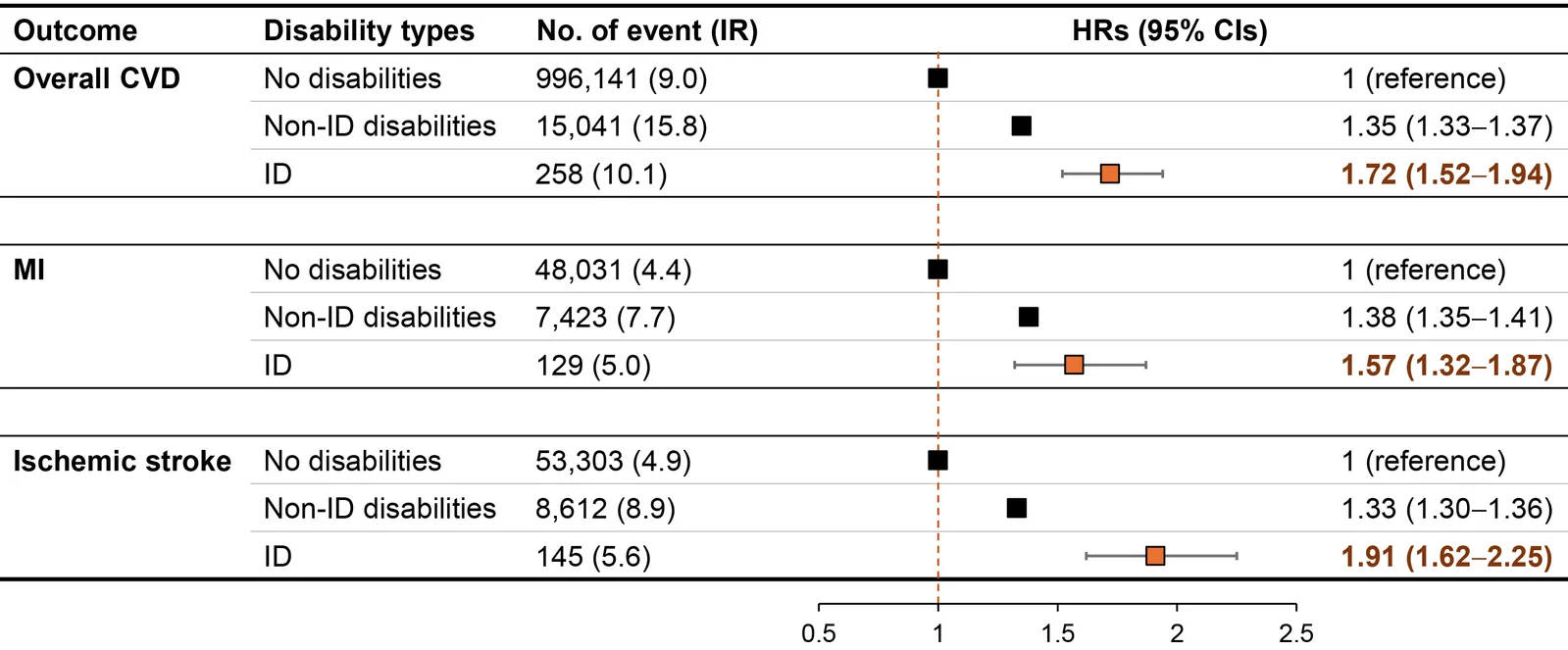
**Risk of incident cardiovascular disease by disability status in individuals with type 2 diabetes.** Hazard ratios (HRs) and 95% confidence intervals (CIs) for incident cardiovascular disease (including myocardial infarction and ischemic stroke) are presented according to disability status, using individuals with no disabilities as the reference group. Estimates were adjusted for age, sex, household income (yes/no for low-income group), and residential location (urban/rural). IR, incident rate (per 1000 person-years); HRs, hazard ratios; CIs, confidence intervals; CVD, cardiovascular disease; ID, intellectual disability; MI, myocardial infarction.

**Fig. 3:**
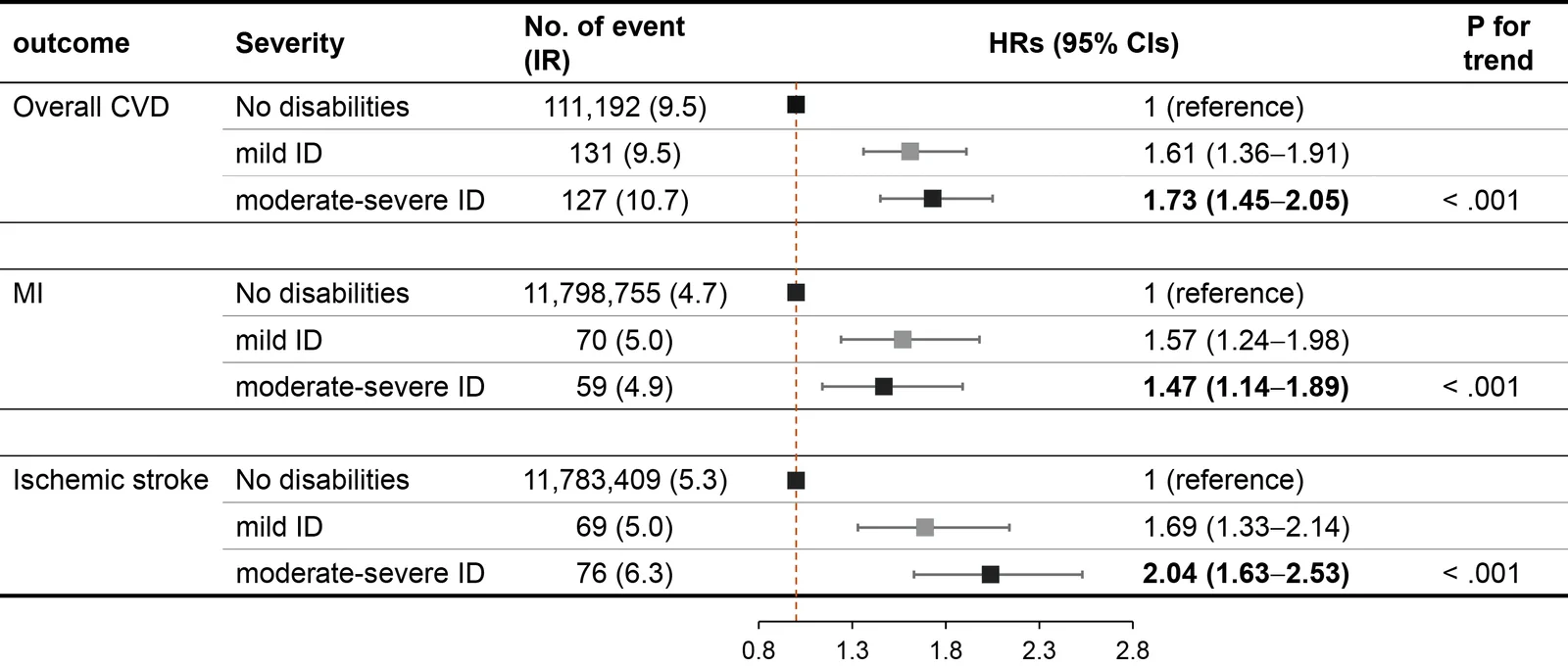
**Association between intellectual disability severity and cardiovascular disease risk among individuals with type 2 diabetes.** Hazard ratios (HRs) and 95% confidence intervals (CIs) for incident cardiovascular disease (including myocardial infarction and ischemic stroke) are presented according to the severity of intellectual disability (ID), using individuals with no disabilities as the reference group. ID severity was categorized into two levels: moderate-to-severe ID (grade 1, IQ < 35; grade 2, IQ 35–49) and mild ID (grade 3, IQ 50–69). Estimates were adjusted for age, sex, household income (yes/no for low-income group), and residential location (urban/rural). IR, incident rate (per 1000 person-years); HRs, hazard ratios; CIs, confidence intervals; P for trend, P-value for linear trend; CVD, cardiovascular disease; ID, intellectual disability; MI, myocardial infarction.

Over a median follow-up of 5.9 (5.3−6.3) years, 111,450 incident CVD events (5.4%) were recorded. The crude incidence rates per 1000 person-years were 10.1 for the ID group, 15.8 for the non-ID disability group, and 9.5 for the no disability group. Compared to the no disability reference group, individuals with ID exhibited a substantially higher adjusted risk of overall CVD (HR 1.72; 95% CI 1.52−1.94), significantly exceeding the risk observed in those with non-ID disabilities (HR 1.35; 95% CI 1.33−1.37; P < .001) (Fig. 2, Supplementary Table S2). For specific CVD subtypes, this heightened vulnerability was evident for MI (HR 1.57; 95% CI 1.32−1.87) and particularly pronounced for ischemic stroke (HR 1.91; 95% CI 1.62−2.25). When stratified by ID severity, risk estimates were higher with greater severity (P for trend <0.001; Fig. 3, Supplementary Table S3), with elevated risk evident even in mild ID (HR 1.61; 95% CI 1.36−1.91).

In the fully adjusted model (Model 5), these associations remained robust. Compared to those with no disabilities, the non-ID disability group exhibited a 28% increased risk of overall CVD (HR 1.28; 95% CI 1.25−1.30). Notably, this risk was substantially amplified in the ID group (HR 1.69; 95% CI 1.49−1.91). This pattern was consistent for both MI and ischemic stroke, supporting a distinct cardiovascular burden (Supplementary Table S4). In a post-hoc competing-risk analysis, these associations were materially unchanged in a Fine–Gray subdistribution hazards model that accounted for all-cause mortality as a competing event (subdistribution HR for overall CVD, 1.64; 95% CI 1.45−1.86) (Supplementary Table S5).

**Fig. 4:**
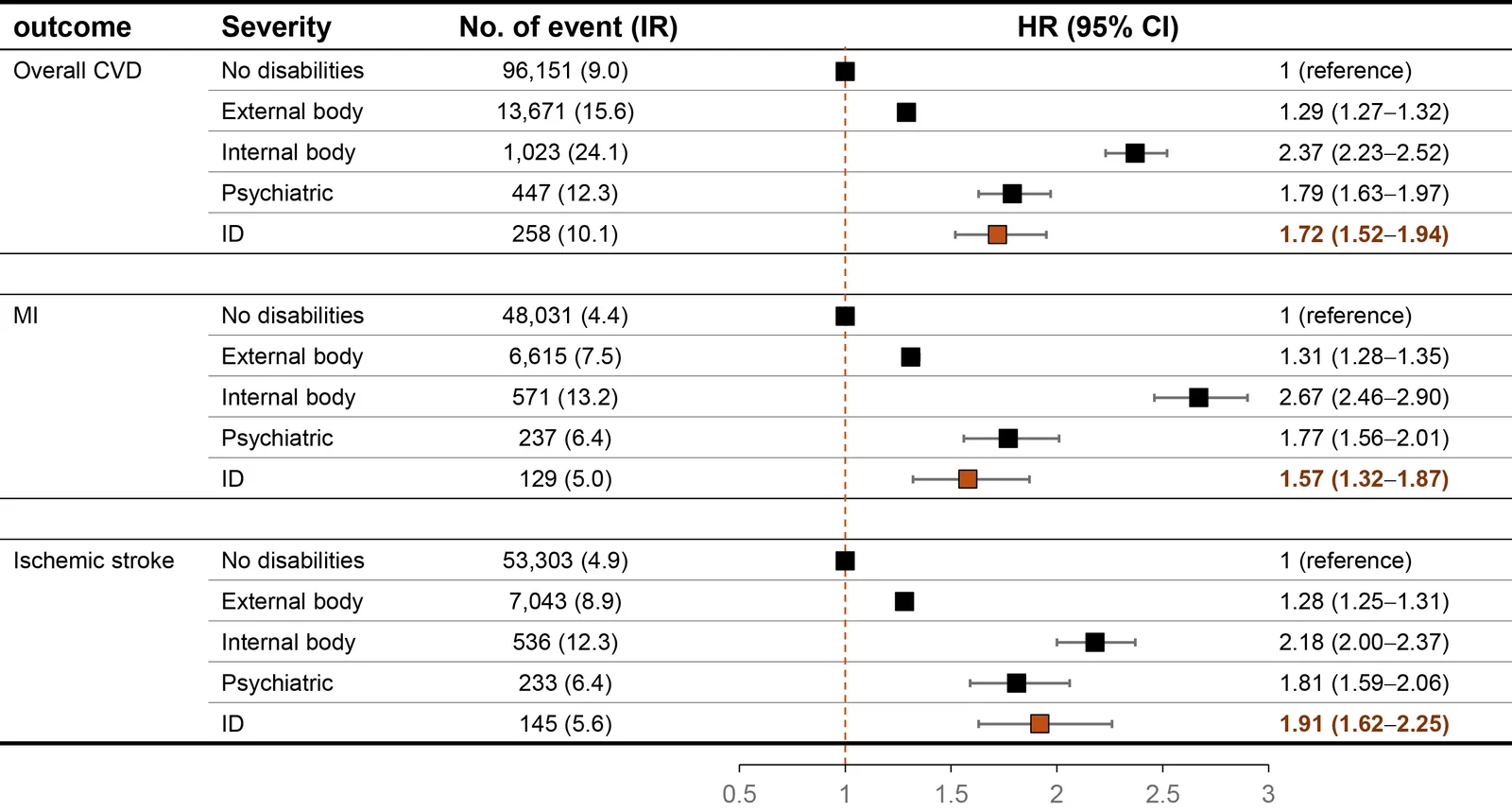
**Association of disability types with cardiovascular disease risk in individuals with type 2 diabetes.** Hazard ratios (HRs) and 95% confidence intervals (CIs) for incident cardiovascular disease (myocardial infarction and ischemic stroke) are presented across four broad disability categories, using individuals with no disabilities as the reference group. The 15 specific disability subtypes were classified as follows: 1) external physical impairments (impairments of the extremities, vision, hearing, speech and language, disabilities due to brain injury, and facial deformities); 2) internal organ dysfunctions (renal failure, heart problems, liver disease, respiratory problems, ostomy, and epilepsy); 3) psychiatric disabilities (autism and mental disorders); and 4) intellectual disability (ID). IR, incident rate (per 1000 person-years); HRs, hazard ratios; CIs, confidence intervals; CVD, cardiovascular disease; ID, intellectual disability; MI, myocardial infarction.

In a sensitivity analysis comparing four broad clinical subgroups, internal organ impairments conferred the highest risk (HR 2.37; 95% CI 2.23−2.52). However, the ID group showed a substantial risk (HR 1.72; 95% CI 1.52−1.94) comparable to psychiatric disabilities (HR 1.79; 95% CI 1.63−1.97) and significantly greater than external physical disabilities (HR 1.29; 95% CI 1.27−1.32), a pattern consistent across CVD subtypes (Fig. 4, Supplementary Table S6). In a post-hoc sensitivity analysis, integrating individuals with autism (n = 38) into the ID group yielded virtually identical, significantly elevated CVD risks (HR 1.71; 95% CI 1.51−1.93) (Supplementary Table S7).

In stratified analyses, the elevated CVD risk among individuals with ID was remarkably consistent across all clinical and sociodemographic strata (Supplementary Table S8). We found no significant effect modification by age (<50 vs. ≥50 years; *P* for interaction = 0.82), income status (*P* for interaction = 0.73), or major comorbidities such as obesity (*P* for interaction = 0.31), hypertension (*P* for interaction = 0.35), and dyslipidemia (*P* for interaction = 0.15). Also, the association was not modified by diabetes-related factors, including diabetes duration (<5 years vs. ≥5 years; P for interaction = 0.18) or insulin use status (*P* for interaction = 0.78).

## Discussion

In this large, nationwide cohort, we found that comorbid ID was associated with a substantial and distinct excess risk of incident CVD among adults with T2D, significantly exceeding the risk associated with other disabilities. While a recent systematic review comprehensively synthesized the broader cardiovascular burden in adults with ID,^25^ our study extends this evidence by focusing on adults with concomitant T2D. By demonstrating amplified CVD incidence that persisted after comprehensive adjustment for cardiometabolic risk factors, our findings suggest that T2D may further increase cardiovascular vulnerability among individuals with ID.

The central finding of our study is that ID was associated with a distinct cardiovascular vulnerability relative to other disabilities. While previous studies have established increased CVD risk among individuals with psychiatric disorders^26^ or physical disabilities,^27^ direct comparisons across disability subtypes remain remarkably scarce. Our study addresses this gap, revealing that the cardiovascular burden associated with ID is substantial, comparable to that of major psychiatric disabilities and markedly higher than that of physical disabilities. These findings reframe the issue from one of general risk identification to the urgent recognition of a significant health disparity.

Consistent with our findings, previous population-based studies from Denmark and the UK have demonstrated elevated CVD risks among younger individuals with ID, specifically, noting a 24% overall increase from early life to mid-adulthood,^16^ and a 12% increase in macrovascular complications with concurrent T2D.^1^ In contrast, studies focusing on older adults often failed to observe such a significant association.^18^^,^^19^ By encompassing a broad age spectrum, our study helps clarify these discrepancies, demonstrating that the elevated CVD risk persists across adulthood. These findings support prioritizing cardiovascular management across the life course for individuals with ID. Furthermore, because early-onset T2D is a potent driver of premature CVD,^28^ our findings suggest that ID is associated with additional cardiovascular burden, even within an already high-risk T2D population.

Although the ID cohort displayed the lowest rates of smoking and heavy alcohol consumption, their concurrent high prevalence of obesity, physical inactivity, and socioeconomic disadvantage suggests that these patterns may reflect systemic constraints rather than health-conscious choices.^29^ Similarly, the lower rates of diagnosed hypertension and dyslipidemia likely reflect the younger age of the ID group, although under-recognition of chronic cardiometabolic conditions in routine clinical care cannot be excluded. The early reliance on complex therapeutic regimens (e.g., insulin or triple-combination therapy) further underscores challenges in self-management and medication adherence. Consequently, the substantially increased CVD risk in this population may be related to cognitive and systemic barriers that impede optimal metabolic control, underscoring the need for proactive, multidisciplinary care models.

Interestingly, the risk profiles for specific cardiovascular events differed within our ID cohort. Consistent with a previous study in older Dutch adults showing higher stroke but lower MI incidence,^30^ our study showed that while MI risk was elevated across ID severity levels, ischemic stroke estimates were higher with greater ID severity. Although overlapping confidence intervals preclude a definitive assertion that severity impacts ischemic stroke more than MI, this cerebrovascular pattern is clinically noteworthy. The heightened risk of ischemic stroke may be related to ID-specific factors, including embolic conditions from congenital heart disease, higher atrial fibrillation rates, and the use of antipsychotic drugs or mood stabilizers.^30^, ^31^, ^32^ Therefore, comprehensive cardiovascular care, particularly with attention to stroke prevention, may be warranted across all severity levels, given that elevated vascular risk was evident even in mild ID.

The primary strengths of our study include its nationwide scope, large sample size, and the novel direct comparison between disability groups, which allowed us to isolate the unique CVD risk associated with ID. However, several limitations warrant consideration. First, because the Korean NDRS relies on specific administrative criteria, variations in diagnostic thresholds may limit the generalizability of our findings to countries with different disability classification systems. Second, our exclusion of individuals with pre-existing CVD to improve temporality, combined with the requirement of attending health screenings, likely underrepresented the most severely affected or underserved individuals with ID. Because these criteria may have biased the sample toward a relatively healthier or better-supported subset, our findings should be interpreted primarily as applying to screening-participating adults with registry-defined ID and T2D, and generalizability to the broader ID population may be limited. Third, despite employing rigorous operational definitions, some misclassification from undiagnosed cases may persist. Such nondifferential misclassification would most likely bias the association toward the null, potentially attenuating the observed association. Fourth, a residual confounding from unmeasured variables, such as medication adherence, psychosocial stress, diet quality, access to healthcare, and early-life factors, is inherent to administrative data. However, the consistency of our results across multiple sensitivity and stratified analyses mitigates this concern. Additionally, while Down syndrome carries a unique stroke-predominant cardiovascular profile,^33^ only 30 individuals with Down syndrome were identified in our cohort, which is consistent with the sharply declining prevalence of Down syndrome in older adults, typical of T2D onset.^34^ Although this small number precluded a separate subgroup analysis, etiologic heterogeneity within the ID group remains a limitation. Fifth, although the median follow-up of 5.9 years is relatively short, the observation of elevated incident CVD risk, particularly ischemic stroke, within this timeframe underscores the clinical relevance of this finding. Future longitudinal studies with extended follow-up are warranted to clarify the long-term cardiovascular trajectory of adults with ID and T2D. Finally, our observational design precludes causal inferences.

Our study demonstrated that individuals with T2D and comorbid ID represent a distinct group with substantially elevated CVD risk, with a risk burden that significantly exceeds that of other disability groups. This marked health disparity, particularly for ischemic stroke and evident even in individuals with mild ID, is not fully explained by conventional risk factors. These findings highlight the need for clinicians to recognize ID as an important marker of cardiovascular vulnerability. Care models may need to be adapted to address the unique needs of this population, incorporating risk factor management and multidisciplinary support to overcome barriers to self-management and mitigate cardiovascular risk.

## Contributors

JHB conceptualized the study, wrote the original draft, and contributed to review and editing. YMMP contributed to data curation, conceptualization, methodology, and review and editing. GEN contributed to investigation and review and editing. OKH contributed to review and editing. EP contributed to review and editing. SHK contributed to review and editing. SSL contributed to conceptualization, visualization, validation, investigation, formal analysis, data curation, supervision, and writing of the original draft, and review and editing. KH contributed to conceptualization, validation, investigation, formal analysis, supervision, and writing of the original draft, and review and editing. KH and SSL accessed and verified the underlying data. SSL had final responsibility for the decision to submit for publication. All authors read and approved the final version of the manuscript.

## Data sharing statement

The data underlying this study were obtained from the Korean National Health Insurance Service (NHIS) and are not publicly available due to legal and privacy restrictions governing the use of national health insurance data in South Korea. The statistical analysis code used in this study is available from the corresponding author upon reasonable request.

## Declaration of interests

All authors declare no competing interests.
