# Supplementary material for: Cardiovascular risk associated with intellectual disability among adults with type 2 diabetes: a nationwide cohort study in South Korea
Source: eClinicalMedicine. 2026 Jul 1;97:104043. doi: 10.1016/j.eclinm.2026.104043 (PMC13352028; doi:10.1016/j.eclinm.2026.104043)
Supplement: Supplementary Material [file mmc1.docx]

Supplementary Appendix

**Cardiovascular Risk Associated with Intellectual disability Among Adults with Type 2 Diabetes: A Nationwide Cohort Study in South Korea**

**Supplementary Methods.** Supplementary methods

**Supplementary Table S1.** Baseline characteristics according to disability types

**Supplementary Table S2.** Disability status and the risk of overall cardiovascular disease and its subtypes among individuals with type 2 diabetes

**Supplementary Table S3.** The severity of intellectual disability and the risk of overall cardiovascular disease and its subtypes among individuals with type 2 diabetes

**Supplementary Table S4.** Disability status and the risk of overall cardiovascular disease and its subtypes in individuals with type 2 diabetes, with further adjustment for lifestyle factors, comorbidities, and diabetes-related factors

**Supplementary Table S5.** Risk of incident cardiovascular disease by disability type, accounting for all-cause mortality as a competing risk (Fine-Gray model)

**Supplementary Table S6.** Types of Disability and the risk of overall cardiovascular diseases and their subtypes among individuals with type 2 diabetes

**Supplementary Table S7.** Association between disability types and the risk of cardiovascular diseases, refining intellectual disability and autism combined as neurodevelopmental disorders

**Supplementary Table S8.** Association between intellectual disability and risk of overall cardiovascular disease and its subtypes in individuals with type 2 diabetes stratified by selected factors

**Supplementary Methods**

Information on lifestyle-related factors was obtained using self-administered questionnaires. Smoking status was categorized as current smoker, ex-smoker, or non-smoker. Alcohol consumption was also categorized into non-drinking, mild to moderate drinking (<30 g/day), and heavy drinking (≥30 g/day).^1^ Low household income status was defined as being in the lowest income quartile based on health insurance premiums or receiving Medical Aid benefits.^2^ For physical activity, regular exercise was defined as at least 30 minutes of moderate physical activity for ≥5 days weekly or at least 20 minutes of strenuous physical activity ≥3 days weekly.^3^ The anthropometric and clinical characteristics were assessed by trained personnel during the health screening examination. Body mass index (BMI) was calculated by weight in kilograms divided by the square of height in meters (kg/m^2^). General obesity was defined as a body mass index (BMI) of ≥25 kg/m^2^. Abdominal obesity was defined as a waist circumference of ≥90 cm for men and ≥85 cm for women, according to Korean population standards and guidelines.^4^ Systolic and diastolic blood pressures were measured after at least 5 minutes of rest with the patient in a sitting position. Blood samples were collected after overnight fasting, and glucose, lipid profiles, and serum creatinine levels were measured. Quality control procedures for laboratory tests were conducted in accordance with the Korean Association of Laboratory Quality Control guidelines.^5^

The presence of hypertension was defined as having at least one prescription of antihypertensive medications under ICD-10 codes (I10–I13, I15) per year or systolic/diastolic blood pressure ≥140/90 mmHg.^6^ Dyslipidemia was defined as a total cholesterol level of ≥240 mg/dL or taking lipid-lowering drugs under the ICD-10 code (E78).^7^ Chronic kidney disease was defined by claims for dialysis with special reimbursement code (V001, V003, and V005) or by a low estimated glomerular filtration rate (<60 mL/min).^8^ Newly diagnosed diabetes was defined as having no history of claims for the ICD-10 code of diabetes (E11-E14) or anti-diabetic medication before baseline, and FPG ≥126 mg/dL at baseline.^9^ Diabetes duration was calculated as the time interval from the earliest recorded date of T2D diagnosis available in the NHIS database since 2002 to the baseline index year.

**Reference)**

1 Dufour MC. What is moderate drinking? Defining ‘drinks’ and drinking levels. *Alcohol Res Health* 1999; **23**: 5–14.

2 Park Y-MM, Baek J-H, Lee HS, *et al.* Income variability and incident cardiovascular disease in diabetes: a population-based cohort study. *Eur Heart J* 2024; **45**: 1920–33.

3 Rosenberg DE, Bull FC, Marshall AL, Sallis JF, Bauman AE. Assessment of sedentary behavior with the International Physical Activity Questionnaire. *J Phys Act Health* 2008; **5 Suppl 1**: S30-44.

4 Seo MH, Lee W-Y, Kim SS, *et al.* 2018 Korean Society for the Study of Obesity Guideline for the Management of Obesity in Korea. *J Obes Metab Syndr* 2019; **28**: 40–5.

5 Lee J, Lee JS, Park S-H, Shin SA, Kim K. Cohort Profile: The National Health Insurance Service–National Sample Cohort (NHIS-NSC), South Korea. *International Journal of Epidemiology* 2017; **46**: e15.

6 Lee H-Y, Shin J, Kim G-H, *et al.* 2018 Korean Society of Hypertension Guidelines for the management of hypertension: part II-diagnosis and treatment of hypertension. *Clin Hypertens* 2019; **25**: 20.

7 Rhee E-J, Kim HC, Kim JH, *et al.* 2018 Guidelines for the management of dyslipidemia. *Korean J Intern Med* 2019; **34**: 723–71.

8 Levin A, Stevens PE. Summary of KDIGO 2012 CKD Guideline: behind the scenes, need for guidance, and a framework for moving forward. *Kidney International* 2014; **85**: 49–61.

9 Ko S-H, Han K, Lee Y, *et al.* Past and Current Status of Adult Type 2 Diabetes Mellitus Management in Korea: A National Health Insurance Service Database Analysis. *Diabetes Metab J* 2018; **42**: 93–100.

**Supplementary Table S1.** Baseline characteristics according to disability types

|  | No disabilities | Disability types | | | |
| --- | --- | --- | --- | --- | --- |
|  |  | External body | Internal body | Psychiatric | ID |
| N | 1,884,897 | 158,442 | 8,367 | 6,541 | 4,574 |
| **Percentage (%)** |  |  |  |  |  |
| Age groups, years |  |  |  |  |  |
| 20 to <40 | 6.1 | 1.4 | 1.9 | 3.2 | 16.2 |
| 40 to <65 | 66.3 | 51.1 | 63.9 | 89.1 | 79.1 |
| ≥65 | 27.5 | 47.6 | 34.2 | 7.7 | 4.7 |
| Sex, male | 61.3 | 63.1 | 69.0 | 47.3 | 54.3 |
| Low-income status, yes* | 20.6 | 27.0 | 29.7 | 74.7 | 78.1 |
| Residential location |  |  |  |  |  |
| Urban | 44.3 | 40.8 | 43.9 | 38.7 | 30.9 |
| Rural | 55.7 | 59.2 | 56.1 | 61.3 | 69.1 |
| Smoking |  |  |  |  |  |
| Non-smoker | 53.2 | 56.0 | 56.6 | 61.0 | 81.4 |
| Ex-smoker | 22.4 | 23.0 | 30.9 | 8.9 | 5.8 |
| Current smoker | 24.4 | 21.0 | 12.4 | 30.2 | 12.8 |
| Alcohol consumption |  |  |  |  |  |
| None | 53.7 | 62.3 | 81.7 | 88.1 | 87.1 |
| Mild | 36.2 | 28.8 | 16.0 | 10.0 | 10.7 |
| Heavy | 10.1 | 9.0 | 2.3 | 1.9 | 2.2 |
| Regular exercise, yes† | 22.1 | 20.4 | 21.6 | 12.8 | 15.0 |
| Duration of diabetes |  |  |  |  |  |
| Newly diagnosed | 34.5 | 23.1 | 12.1 | 27.1 | 32.6 |
| <5 years | 24.3 | 22.9 | 19.1 | 29.9 | 27.9 |
| 5 to <10 years | 19.8 | 23.5 | 22.9 | 28.0 | 25.1 |
| ≥10 years | 21.3 | 30.5 | 46.0 | 15.0 | 14.3 |
| Insulin prescription, yes | 7.4 | 11.5 | 39.8 | 9.8 | 9.4 |
| Use of triple-combination drugs, yes | 22.2 | 25.4 | 15.7 | 24.3 | 24.5 |
| Hypertension, yes | 53.4 | 64.0 | 71.9 | 40.6 | 44.6 |
| Dyslipidemia, yes | 54.3 | 56.9 | 52.4 | 54.4 | 47.2 |
| Obesity, yes | 51.5 | 51.5 | 36.1 | 61.9 | 54.4 |
| CKD, yes | 7.4 | 12.5 | 63.7 | 9.2 | 5.5 |
| **Mean (SD)** |  |  |  |  |  |
| Age, years | 57.6 ± 11.8 | 63.7 ± 10.6 | 60.5 ± 10.2 | 53.4 ± 8.5 | 48.7 ± 10.6 |
| BMI, kg/m^2^ | 25.4 ± 3.6 | 25.3 ± 3.6 | 24.0 ± 3.5 | 26.5 ± 4.4 | 25.9 ± 4.8 |
| Blood pressure, mm Hg |  |  |  |  |  |
| Systolic | 128 ± 15 | 129 ± 15 | 130 ± 17 | 122 ± 15 | 125 ± 16 |
| Diastolic | 78 ± 10 | 78 ± 10 | 77 ± 10 | 77 ± 10 | 78 ± 11 |
| FPG, mg/dL | 146 ± 46 | 141 ± 45 | 135 ± 48 | 145 ± 55 | 149 ± 65 |
| Total cholesterol, mg/dL | 189 ± 44 | 182 ± 42 | 171 ± 40 | 184 ± 46 | 186 ± 44 |
| LDL-cholesterol, mg/dL | 106 ± 39 | 101 ± 37 | 93 ± 34 | 103 ± 39 | 105 ± 38 |

Abbreviations: ID, intellectual disability; CKD, chronic kidney disease; SD, standard deviations; FPG, fasting plasma glucose.

*Low household income status was defined as being in the lowest quartile of monthly income based on health insurance premiums or receiving benefits under the Medical Aid program

† Regular exercise was defined to be at least 30 minutes of moderate physical activity for ≥5 days weekly or at least 20 minutes of strenuous physical activity ≥3 days weekly

**Supplementary Table S2.** Disability status and the risk of overall cardiovascular disease and its subtypes among individuals with type 2 diabetes

| Outcomes | Type of disability | Subjects  (N) | Events  (N) | PY of follow-up | IR (per  1,000 PY) | **HRs (95% CIs) *** | | |
| --- | --- | --- | --- | --- | --- | --- | --- | --- |
|  |  |  |  |  |  | Model 1 | Model 2 | Model 3 |
| **Overall CVD** | No disabilities | 1,884,897 | 96,151 | 10,710,721 | 9.0 | 1 (reference) | 1 (reference) | 1 (reference) |
|  | Non-ID disabilities | 173,350 | 15,041 | 949,838 | 15.8 | 1.77 (1.74–1.80) | 1.36 (1.34–1.39) | 1.35 (1.33–1.37) |
|  | ID | 4,574 | 258 | 25,676 | 10.1 | 1.12 (0.99–1.26) | 1.88 (1.66–2.12) | 1.72 (1.52–1.94) |
|  | *P-value* |  |  |  |  | <0.001 | <0.001 | < .001 |
| **MI** | No disabilities | 1,884,897 | 48,031 | 10,830,503 | 4.4 | 1 (reference) | 1 (reference) | 1 (reference) |
|  | Non-ID disabilities | 173,350 | 7,423 | 968,252 | 7.7 | 1.73 (1.69–1.77) | 1.39 (1.36–1.43) | 1.38 (1.35–1.41) |
|  | ID | 4,574 | 129 | 25,960 | 5.0 | 1.12 (0.94–1.33) | 1.70 (1.43–2.02) | 1.57 (1.32–1.87) |
|  | *P-value* |  |  |  |  | <0.001 | <0.001 | < .001 |
| **Ischemic stroke** | No disabilities | 1,884,897 | 53,303 | 10,817,766 | 4.9 | 1 (reference) | 1 (reference) | 1 (reference) |
|  | Non-ID disabilities | 173,350 | 8,612 | 965,644 | 8.9 | 1.81 (1.77–1.85) | 1.34 (1.31–1.37) | 1.33 (1.30–1.36) |
|  | ID | 4,574 | 145 | 25,933 | 5.6 | 1.13 (0.96–1.34) | 2.11 (1.79–2.49) | 1.91 (1.62–2.25) |
|  | *P-value* |  |  |  |  | <0.001 | <0.001 | < .001 |

Abbreviation: IR, incident rates; PY, person-year; HRs, hazard ratios; CIs, confidence intervals; CVD, cardiovascular disease; ID, intellectual disability; MI, myocardial infarction.

*** Model 1:** unadjusted**. Model 2:** adjusted for age and sex. **Model 3 (primary)**: adjusted for age, sex, household income (yes/no for low-income group), residential location (urban/rural)

**Supplementary Table S3.** The severity of intellectual disability and the risk of overall cardiovascular disease and its subtypes in individuals with type 2 diabetes

| Outcomes | Severity of ID | Subjects  (N) | Events  (N) | PY of follow-up | IR (per  1,000 PY) | **HRs (95% CIs) *** | | |
| --- | --- | --- | --- | --- | --- | --- | --- | --- |
|  |  |  |  |  |  | Model 1 | Model 2 | Model 3 |
| **Overall CVD** | No | 2,058,247 | 111,192 | 11,660,559 | 9.5 | 1 (reference) | 1 (reference) | 1 (reference) |
|  | Mild ID | 2,451 | 131 | 13,761 | 9.5 | 1.00 (0.84–1.18) | 1.79 (1.51–2.12) | 1.61 (1.36–1.91) |
|  | Moderate to severe ID | 2,123 | 127 | 11,915 | 10.7 | 1.13 (0.95–1.34) | 1.88 (1.58–2.23) | 1.73 (1.45–2.05) |
|  | *P for trend* |  |  |  |  | <.0001 | <.0001 | < .001 |
| **MI** | No | 2,058,247 | 55,454 | 11,798,755 | 4.7 | 1 (reference) | 1 (reference) | 1 (reference) |
|  | Mild ID | 2,451 | 70 | 13,884 | 5.0 | 1.08 (0.85–1.36) | 1.73 (1.37–2.18) | 1.57 (1.24–1.98) |
|  | Moderate to severe ID | 2,123 | 59 | 12,075 | 4.9 | 1.04 (0.81–1.34) | 1.59 (1.23–2.05) | 1.47 (1.14–1.89) |
|  | *P for trend* |  |  |  |  | <.0001 | <.0001 | < .001 |
| **Ischemic stroke** | No | 2,058,247 | 61,915 | 11,783,409 | 5.3 | 1 (reference) | 1 (reference) | 1 (reference) |
|  | Mild ID | 2,451 | 69 | 13,908 | 5.0 | 0.94 (0.745–1.20) | 1.89 (1.49–2.40) | 1.69 (1.33–2.14) |
|  | Moderate to severe ID | 2,123 | 76 | 12,025 | 6.3 | 1.20 (0.96–1.51) | 2.24 (1.79–2.81) | 2.04 (1.63–2.56) |
|  | *P for trend* |  |  |  |  | <.0001 | <.0001 | < .001 |

Abbreviation: ID, intellectual disability; IR, incident rates; PY, person-year; HRs, hazard ratios; CIs, confidence intervals; CVD, cardiovascular disease; ID, intellectual disability; MI, myocardial infarction.

*** Model 1:** unadjusted**. Model 2:** adjusted for age and sex. **Model 3 (primary)**: adjusted for age, sex, household income (yes/no for low-income group), residential location (urban/rural)

**Supplementary Table S4.** Disability status and the risk of overall cardiovascular disease and its subtypes in individuals with type 2 diabetes, with further adjustment for lifestyle factors, comorbidities, and diabetes-related factors

| Outcomes | ID | **HRs (95% CIs)**† | | | | |
| --- | --- | --- | --- | --- | --- | --- |
|  |  | Model 1 | Model 2 | **Model 3 (primary)** | Model 4 | Model 5 |
| **Overall CVD*** | No disabilities | 1 (reference) | | | | |
|  | Non-ID disabilities | 1.77 (1.74–1.80) | 1.36 (1.34–1.39) | 1.35 (1.33–1.37) | 1.33 (1.31–1.35) | 1.28 (1.25–1.30) |
|  | ID | 1.12 (0.99–1.26) | 1.88 (1.66–2.12) | 1.72 (1.52–1.94) | 1.71 (1.52–1.94) | 1.69 (1.49–1.91) |
|  | *P-value* | < .001 | < .001 | < .001 | < .001 | < .001 |
| **MI** | No disabilities | 1 (reference) | | | | |
|  | Non-ID disabilities | 1.73 (1.69–1.77) | 1.39 (1.36–1.43) | 1.38 (1.35–1.41) | 1.35 (1.32–1.39) | 1.29 (1.26–1.32) |
|  | ID | 1.12 (0.94–1.33) | 1.70 (1.43–2.02) | 1.57 (1.32–1.87) | 1.53 (1.28–1.82) | 1.51 (1.27–1.80) |
|  | *P-value* | < .001 | < .001 | < .001 | < .001 | < .001 |
| **Ischemic stroke** | No disabilities | 1 (reference) | | | | |
|  | Non-ID disabilities | 1.81 (1.77–1.85) | 1.34 (1.31–1.37) | 1.33 (1.30–1.36) | 1.31 (1.28–1.34) | 1.27 (1.24–1.30) |
|  | ID | 1.13 (0.96–1.34) | 2.11 (1.79–2.49) | 1.91 (1.62–2.25) | 1.95 (1.66–2.30) | 1.91 (1.63–2.25) |
|  | *P-value* | < .001 | < .001 | < .001 | < .001 | < .001 |

Abbreviation: ID, intellectual disability; HRs, hazard ratios; CIs, confidence intervals; CVD, cardiovascular disease; MI, myocardial infarction.

* Overall CVD outcomes include myocardial infarction and ischemic stroke

† **Model 1:** unadjusted

**Model 2:** adjusted for age and sex

**Model 3 (primary):** adjusted for age, sex, household income (yes/no for low-income group), and residential location (urban/rural)

**Model 4:** adjusted for Model 3 covariates in addition to lifestyle factors (alcohol consumption, smoking, and physical activity)

**Model 5 (fully adjusted):** adjusted for Model 4 covariates in addition to comorbidities (obesity, hypertension, dyslipidemia, chronic kidney disease) and diabetes-related factors (diabetes duration, insulin use, polypharmacy, fasting plasma glucose)

**Supplementary Table S5.** Risk of incident cardiovascular disease by disability type, accounting for all-cause mortality as a competing risk (Fine-Gray model)

| Type of disability* | **SHRs with 95% CIs†** | | |
| --- | --- | --- | --- |
|  | **Overall CVD** | **MI** | **Ischemic stroke** |
| No disabilities | 1 (reference) | | |
| Non-ID disabilities | 1.33 (1.30-1.35) | 1.35 (1.32-1.38) | 1.31 (1.28-1.34) |
| ID | 1.64 (1.45-1.86) | 1.51 (1.27-1.79) | 1.81 (1.54-2.14) |
| *P-value* | < .001 | < .001 | < .001 |

Abbreviations: SHRs, subdistribution hazard ratios; CIs, confidence intervals; CVD, cardiovascular disease; MI, myocardial infarction; ID, intellectual disability

† **Model 3 (primary)**: adjusted for age, sex, household income (yes/no for low-income group), residential location (urban/rural)

**Supplementary Table S6.** Types of Disability and the risk of overall cardiovascular diseases and their subtypes among individuals with type 2 diabetes

| Outcomes | Type of disability * | Subjects  (N) | Events  (N) | PY of follow-up | IR (per  1,000 PY) | **HRs (95% CIs)** † | | |
| --- | --- | --- | --- | --- | --- | --- | --- | --- |
|  |  |  |  |  |  | Model 1 | Model 2 | Model 3 |
| **Overall CVD** | No | 1,884,897 | 96,151 | 10,710,721 | 9.0 | 1 (reference) | 1 (reference) | 1 (reference) |
|  | External body | 158,442 | 13,571 | 871,153 | 15.6 | 1.74 (1.71–1.77) | 1.31 (1.28–1.33) | 1.29 (1.27–1.32) |
|  | Internal body | 8,367 | 1,023 | 42,404 | 24.1 | 2.71 (2.55–2.88) | 2.39 (2.25–2.54) | 2.37 (2.23–2.52) |
|  | Psychiatric other than ID | 6,541 | 447 | 36,281 | 12.3 | 1.37 (1.25–1.51) | 1.93 (1.76–2.12) | 1.79 (1.63–1.97) |
|  | ID | 4,574 | 258 | 25,676 | 10.1 | 1.12 (0.99–1.26) | 1.88 (1.66–2.13) | 1.72 (1.52–1.95) |
|  | *P-value* |  |  |  |  | < .001 | < .001 | < .001 |
| **MI** | No | 1,884,897 | 48,031 | 10,830,503 | 4.4 | 1 (reference) | 1 (reference) | 1 (reference) |
|  | External body | 158,442 | 6,615 | 888,094 | 7.5 | 1.68 (1.64–1.72) | 1.32 (1.29-1.36) | 1.31 (1.28–1.35) |
|  | Internal body | 8,367 | 571 | 43,359 | 13.2 | 2.99 (2.76–3.25) | 2.70 (2.48-2.93) | 2.67 (2.46–2.90) |
|  | Psychiatric other than ID | 6,541 | 237 | 36,799 | 6.4 | 1.45 (1.28–1.65) | 1.90 (1.67–2.16) | 1.77 (1.56–2.01) |
|  | ID | 4,574 | 129 | 25,960 | 5.0 | 1.12 (0.94–1.33) | 1.71 (1.44–2.03) | 1.58 (1.32–1.87) |
|  | *P-value* |  |  |  |  | < .001 | < .001 | < .001 |
| **Ischemic stroke** | No | 1,884,897 | 53303 | 10,817,766 | 4.9 | 1 (reference) | 1 (reference) | 1 (reference) |
|  | External body | 158,442 | 7843 | 885,425 | 8.9 | 1.80 (1.76–1.84) | 1.29 (1.26–1.33) | 1.28 (1.25–1.31) |
|  | Internal body | 8,367 | 536 | 43,500 | 12.3 | 2.52 (2.31–2.74) | 2.20 (2.02–2.39) | 2.18 (2.00–2.37) |
|  | Psychiatric other than ID | 6,541 | 233 | 36,719 | 6.4 | 1.29 (1.13–1.46) | 1.96 (1.72–2.23) | 1.81 (1.59–2.06) |
|  | ID | 4,574 | 145 | 25,933 | 5.6 | 1.13 (0.96–1.34) | 2.12 (1.80–2.49) | 1.92 (1.63–2.26) |
|  | *P-value* |  |  |  |  | < .001 | < .001 | < .001 |

Abbreviations: IR, incident rate; PY, person-year; HRs, hazard ratios; CIs, confidence intervals; CVD, cardiovascular disease; ID, intellectual disability; MI, myocardial infarction

* Types of disability were defined by the Korean national disability registration system. **External body disabilities** comprise disability of the extremities due to brain injury; visual, hearing, speech and language, and facial deformity disability. **Internal body disabilities** comprise disability due to renal failure, heart, liver, respiratory, ostomy, and epilepsy. **Psychiatric disabilities other than ID** comprise disabilities due to autism and mental disorders.

† **Model 1:** unadjusted**. Model 2:** adjusted for age and sex. **Model 3 (primary)**: adjusted for age, sex, household income (yes/no for low-income group), residential location (urban/rural)

**Supplementary Table S7.** Association between disability types and the risk of cardiovascular diseases, refining intellectual disability and autism combined as neurodevelopmental disorders

| Type of disability* | **HRs with 95% CIs†** | | |
| --- | --- | --- | --- |
|  | **Overall CVD** | **MI** | **Ischemic stroke** |
| No disabilities | 1 (reference) | | |
| Other disabilities | 1.35 (1.32-1.37) | 1.37 (1.34-1.41) | 1.33 (1.30-1.36) |
| ID + autism | 1.71 (1.51-1.93) | 1.57 (1.32-1.87) | 1.90 (1.61-2.23) |
| *P-value* | < .001 | < .001 | < .001 |

Abbreviations: HRs, hazard ratios; CIs, confidence intervals; CVD, cardiovascular disease; MI, myocardial infarction; ID, intellectual disability

* Types of disability were defined by the Korean national disability registration system.

**Neurodevelopmental disorders** comprise disabilities due to intellectual disability and autism.

† **Model 3 (primary)**: adjusted for age, sex, household income (yes/no for low-income group), residential location (urban/rural)

**Supplementary Table S8.** Association between intellectual disability and risk of overall cardiovascular disease and its subtypes in individuals with type 2 diabetes stratified by selected factors

|  | **Overall CVD** | |  | **MI** | |  | **Ischemic stroke** | |
| --- | --- | --- | --- | --- | --- | --- | --- | --- |
| Subgroup | HRs with 95% CIs* | *P* for interaction |  | HRs with 95% CIs* | *P* for interaction |  | HRs with 95% CIs* | *P* for interaction |
| Age strata |  | 0.82 |  |  | 0.65 |  |  | 0.72 |
| <50 years | 1.62 (1.30–2.03) |  |  | 1.43 (1.06–1.93) |  |  | 1.95 (1.43–2.66) |  |
| ≥50 years | 1.67 (1.44–1.94) |  |  | 1.55 (1.26–1.92) |  |  | 1.82 (1.50–2.21) |  |
| Sex |  | 0.99 |  |  | 0.16 |  |  | 0.72 |
| Male | 1.67(1.42–1.95) |  |  | 1.68 (1.35–2.09) |  |  | 1.95 (1.43–2.66) |  |
| Female | 1.66 (1.37–2.02) |  |  | 1.30 (0.97–1.73) |  |  | 1.82 (1.50–2.21) |  |
| Low-income status† |  | 0.73 |  |  | 0.98 |  |  | 0.89 |
| No | 1.73 (1.35–2.23) |  |  | 1.51 (1.04–2.19) |  |  | 1.90 (1.36–2.66) |  |
| Yes | 1.65 (1.43–1.89) |  |  | 1.52 (1.25–1.85) |  |  | 1.84 (1.53–2.22) |  |
| Obesity |  | 0.31 |  |  | 0.39 |  |  | 0.56 |
| No | 1.56 (1.30–1.86) |  |  | 1.40 (1.08–1.81) |  |  | 1.77 (1.40–2.23) |  |
| Yes | 1.77 (1.50–2.09) |  |  | 1.63 (1.29–2.06) |  |  | 1.95 (1.55–2.45) |  |
| History of smoking |  | 0.17 |  |  | 0.11 |  |  | 0.50 |
| none | 1.86 (1.62–2.12) |  |  | 1.74 (1.44–2.09) |  |  | 2.04 (1.70–2.44) |  |
| ex- or current | 1.47 (1.09–2.00) |  |  | 1.16 (0.73–1.84) |  |  | 1.77 (1.21–2.58) |  |
| Hypertension |  | 0.35 |  |  | 0.39 |  |  | 0.49 |
| No | 1.78 (1.49–2.12) |  |  | 1.65 (1.29–2.11) |  |  | 1.98 (1.56–2.53) |  |
| Yes | 1.58 (1.34–1.87) |  |  | 1.41 (1.11–1.80) |  |  | 1.77 (1.42–2.21) |  |
| Dyslipidemia |  | 0.15 |  |  | 0.93 |  |  | 0.09 |
| No | 1.52 (1.27–1.81) |  |  | 1.51 (1.19–1.93) |  |  | 1.61 (1.27–2.03) |  |
| Yes | 1.82 (1.53–2.15) |  |  | 1.54 (1.20–1.96) |  |  | 2.12 (1.69–2.66) |  |
| Diabetes duration |  | 0.18 |  |  | 0.60 |  |  | 0.25 |
| <5 years | 1.52 (1.27–1.81) |  |  | 1.43 (1.12–1.83) |  |  | 1.67 (1.32–2.12) |  |
| ≥5 years | 1.79 (1.51–2.12) |  |  | 1.57 (1.23–2.01) |  |  | 2.03 (1.62–2.53) |  |
| Insulin use |  | 0.78 |  |  | 0.94 |  |  | 0.90 |
| No | 1.82 (1.59–2.08) |  |  | 1.50 (1.24–1.81) |  |  | 1.83 (1.53–2.18) |  |
| Yes | 1.74 (1.26–2.40) |  |  | 1.48 (0.95–2.29) |  |  | 1.88 (1.24–2.86) |  |

Abbreviations: CVD, cardiovascular disease; MI, myocardial infarction; HRs, hazard ratios; CIs, confidence intervals

* adjusted for age, sex, household income (yes/no for low-income group), residential location (urban/rural) [Model 3]

† Low household income status was defined as being in the lowest quartile of monthly income based on health insurance premiums or receiving benefits under the Medical Aid program
